# Collaborative Care Models to Address Late-Life Depression: Lessons for Low-And-Middle-Income Countries

**DOI:** 10.3389/fpsyt.2015.00064

**Published:** 2015-05-05

**Authors:** Thamara Tapia-Muñoz, Franco Mascayano, Josefina Toso-Salman

**Affiliations:** ^1^Instituto de Nutrición y Tecnología de los Alimentos (INTA), Universidad de Chile, Santiago, Chile; ^2^Escuela de Salud Pública, Facultad de Medicina, Universidad de Chile, Santiago, Chile; ^3^Teachers College, Columbia University, New York, NY, USA

**Keywords:** collaborative care, late-life depression, treatment response, low-middle income, elderly depression

## Introduction

Late-life depression is a disorder specific to the elderly population with very specific characteristics and prognosis (1, 2). In recent years, depressive disorders have been considered a public health concern due to its high prevalence and wide range of disabilities (3–6) Particularly, evidence from research have reported an association between depressive disorders, cognitive impairment, dementia, and suicide in elderly people. Moreover, research findings support the relationship between depressive disorders, low functionality, and poor quality of life within this population (2, 3, 7–9).

Incidence of late-life depression varies across countries. Rates often vary depending on the setting where this syndrome is evaluated. Djernes (10) has reported that in Canadian communities, the recurrence rate of depression in elderly people was <1%, which contrasts from the 42% reported among American nursing home patients. In a survey administered in both urban and rural locations throughout Peru, Mexico, and Venezuela (11), the prevalence of major depressive reported in older adults ranged from 26 to 31%. Lastly, the “Health, Wellbeing, and Aging” survey in Chile (12) found a prevalence of depression at 27% in older adults.

Depression among the geriatric population often goes undetected and undiagnosed. The main reason for this pertains to how dissimilar the clinical presentation is in the elderly population compared to the adult population (13–17). This implies that the elderly often do not meet criteria for a diagnosis of major depression. As a consequence, they often do not receive the appropriate or timely health treatment for their symptoms.

This is further complicated by the fact that older people diagnosed with depression usually do not use mental health services. According to the Global Action on Aging report (18), only half of the seniors population who are diagnosed with a mental illness receive treatment from health-care services (which is principally primary care), and only a fraction of those actually receive specific interventions from these services. Because older adults typically consult, assist, and prefer primary care; these settings are often the most convenient places to diagnosis and treat late-life depression. However, it is often the case that the health-care staff is not properly trained to treat this population.

On the other hand, there is consensus regarding the strategies to properly address late-life depression. According to researchers, first-line treatment for both mild and severe geriatric depression should be a combination of both antidepressants (especially ISRS) and psychotherapy (7, 17, 19) in order to be effective. Recent studies have reported improvement rates with antidepressants to be about 33% responses to treatment varied between 44 and 48% (20, 21). Nevertheless, even though medical treatment contributes significantly to the treatment of depression, only 30–40% of depressed older adults actually adhere to their medication (18).

Aside from psychopharmacological treatments, psychological treatments have also proven. In a meta-analysis by Cuijpers et al. (22), results indicated that having at least 6 months of psychotherapy were effective in reducing late-life depression. Specifically, some useful approaches to psychotherapy include cognitive behavioral therapy, life review therapy, and problem-solving therapy.

The current implementations of these treatments are far from ideal. This is especially in low-and-middle-income countries (LMICs), where there exists a “gap of treatment” concerning mental health. This refers to the discrepancy between the amounts of people who need healthcare with those who actually receive care (23). The World Health Organization (WHO) has reported that the treatment gap involves 76–90% of adult population in developing countries. Considering the complex and unique characteristics of late-life depression, this gap might be even larger in elderly population.

Consequently, collaborative and community care models have been developed to bridge the gaps and to improve the quality of depression care for older people in high-income countries (HICs). These kinds of interventions have been effective in improving treatment adherence and depressive outcomes in HICs (24). These have been particularly constructive for minority populations and individuals living below poverty levels. As such, receiving valuable input and lessons from there members may prove useful for the implementation of future programs in contexts with limited resources.

## Collaborative Care Models to Improve Primary Care in Treatment to Late-Life Depression

For over a decade, collaborative programs have been established in US, United Kingdom, Canada, and Australia. Considering the quality of the evidence reported, the main programs include: (a) IMPACT, (b) PROSPECT, (c) Program to Encourage Active, Rewarding Lives for Seniors (PEARLS), and (d) HEALTHY IDEAS. Overviews of these interventions are presented below.

### Improving mood promoting access to collaborative treatment (IMPACT project)

Improving Mood Promoting Access to Collaborative Treatment is a collaborative and paced care program to late-life depression developed by Uñützer et al. (25). The main goal of this initiative was to improve depression treatment by supporting the use of antidepressant medications and/or providing a course of Problem-Solving Treatment for Primary Care (PST-PC).

The intervention integrates the following components: (1) psycho-education about late-life depression; (2) case management for 12 months conducted by a depression clinical specialist (DCS), psychologists, and nurses; (3) coordination with a regular primary care provider; (4) antidepressant management; and (4) regular PST-PC. The Hopkins Symptom Checklist (HSCL)-20 was used to measure the number of depression-free days within the 24-month follow-up (26).

Follow-up results from Unutzer et al.’s (26) randomized controlled trial (RCT) showed that the intervention group reported significantly less depression severity than the usual care group. At 12 months, the complete remission rate was estimated at 25% compared to 8.3% among the control group. Participants also reported better adherence to antidepressants, as well as an improvement in quality of life and treatment satisfaction.

### Prevention of suicide in primary care elderly (PROSPECT)

PROSPECT consists of a multisite RCT, which tested the impact of a primary care-based intervention aimed at reducing major risk factors for suicide in late life. The project considered depression among the elderly as the principal risk factor for suicide (27). The trial was conducted in 20 primary care centers in urban, suburban, and rural areas in New York City, Philadelphia, and Pittsburgh (7). The intervention was based on a clinical algorithm for treating geriatric depression in a primary care setting.

Fifteen care managers were enrolled in this study, including social workers, nurses, and psychologists. They participated in weekly supervisions and monthly interpersonal therapy sessions. The care managers monitored areas such as treatment adherence, pharmacological side-effects, and providing follow-up care. Remission was defined as the first occurrence of achieving a score of <10 according to the Hamilton Depression Scale.

The main finding was a significant decrease of suicidal rates in the intervention group (29.4–16.5%) compared with usual care group (20.1–17.1%) (27, 28).

### Program to encourage active, rewarding lives for seniors

The goal of the PEARLS program was to simultaneously reduce symptoms of depression while improving the quality of life among the elderly with diagnoses of minor depression and dysthymia. The major components of PEARLS consisted of the inclusion of care managers and problem-solving treatment for primary care (PST-PC). Social, physical, and pleasant activities were also integrated into the program.

At the 6-month assessment, findings demonstrated more than a 50% reduction of in the HSCL-20 depression score for the intervention group (8%) compared to controls (54%). At the 12-month assessment, these results were maintained. Complete remission of depression symptoms was also superior in the intervention group in the follow-up (29).

### Identifying depression, empowering activities for seniors (HEALTHY IDEAS)

Healthy Ideas is a program inspired and developed from IMPACT and PEARLS. It was implemented through a case-management intervention at three social-service agencies. The program was designed to detect and reduce depressive symptoms among older adults with chronic health conditions and functional limitations (30) by collaborating with available community-based services.

According to Casado et al. (31), Healthy IDEAS includes the following main components: (a) screening and assessment, (b) psycho-education, (c) referral and linkage, and (d) behavioral activation. This also includes at least three face-to-face visits and multiple telephone contacts during 3–6 months.

Efficacy of Healthy IDEAS was evaluated through a RCT, which showed a reduction of depression severity and pain, and an increase in activity levels (30). The trial also included an analysis of the interactions among participants, as well as feedback from key stakeholders, case managers, clients, program leaders, and coaches. Most case managers regarded the program as feasible to implement (31).

In sum, the interventions described above have reported promising results in decreasing the burden associated with late-life depression. There is solid evidence regarding the usefulness of approaches such as psycho-education and case management to treat the older population. It may also be possible to implement these “key strategies” in developing countries, taking into account previous successful experiences across communities with mental health models in LMICs (32). Therefore, the feasibility of these interventions will depend mostly on the existence of preceding mental health policies, plans, and programs.

## The Experience of Chile

Launched in 2001, the Chilean Mental Health and Psychiatry Plans recommend the implementation of familiar and community mental health programs. As a result, several interventions concerning mental health promotion, prevention, treatment, and rehabilitation have been put into action in the last 15 years (33), including the introduction of a Program of Treatment for Depression in Primary Health Care (PTDPHC) in 2003 (34). This national strategy incorporated protocols to detect and treat depression for people over 15 years old in the national health-care system. This program relies on a national and integrated network between each levels of care into the health services (especially among first and second level of care). The approaches to treatment consist of psycho-education, counseling, psychotherapy and, depending on severity, pharmacology as well. This strategy is incorporated into the Regime of Explicit Health Guarantees (34) – one of the principal improvements of the Chilean Health Reform (32) – which offers access, quality, opportunity, and financial support to coverage a list of priority diseases including depression.

Initiatives such as the Older People National Health Program are being currently implemented with an emphasis on prevention of dysfunction among the elderly. The delivery of this program involves a screening exam to detect any conditions affecting functionality, as well as case-management delivery to support elders in their communities. Antidepressants (ISRSS) are prescribed as first line of treatment in conjunction with psychotherapy, counseling, and psycho-education.

The effectiveness of the Older People National Health Program has not yet been evaluated but has shown positive initial findings regarding the impact of the Depression National Primary Care Program. For instance, Alvarado and Rojas (35) have reported significant improvements in both depressive and anxious symptoms. However, these analyses did not report specific outcomes to older people with depression.

Some of the limitations found in this study were (a) a tendency for PHC physicians to underestimate the intensity of depressive symptoms; (b) the standard use of treatments according to the severity of the clinical profiles that are not tailored to specific populations; and (c) high rates of treatment dropout (35, 36).

## Conclusion

We reviewed the most important collaborative care models to address late-life depression. All these programs have reported positive results in decreasing depression symptoms and improving adherence to treatment and health service satisfaction.

The feasibility of the implementation of these interventions will depend mostly on the foundation of the mental health community’s policies and plans. In this case, Chile becomes in a strong candidate to integrate a collaborative model to improve the elderly assistance. This could be also applied to countries such as Brazil or Panama (32).

The cost of the implementation has been identified as the major barrier of collaborative care models. However, even in settings with less health-care resources; the programs discussed here might provide valuable insights. For instance, the use of psycho-education and care managers for both late-life depression and comorbid primary care conditions are two among feasible strategies to transfer and include in LMICs (37). Further, we strongly recommend using the task-shifting model for delivering these treatments. This approach has an extensive trajectory in resource-poor contexts with extraordinary results in diminishing treatment gaps and enhancing quality of care (32). Consequently, health-care services through a task-shifting delivery would benefice to elderly people with depression in LMICs.

## Conflict of Interest Statement

The authors declare that the research was conducted in the absence of any commercial or financial relationships that could be construed as a potential conflict of interest.
